# Supplementary figures and images for: Biochemical profile and in vitro biological activities of extracts from seven folk medicinal plants growing wild in southern Tunisia
Source: PLoS One. 2019 Sep 17;14(9):e0213049. doi: 10.1371/journal.pone.0213049 (PMC6748424; doi:10.1371/journal.pone.0213049)

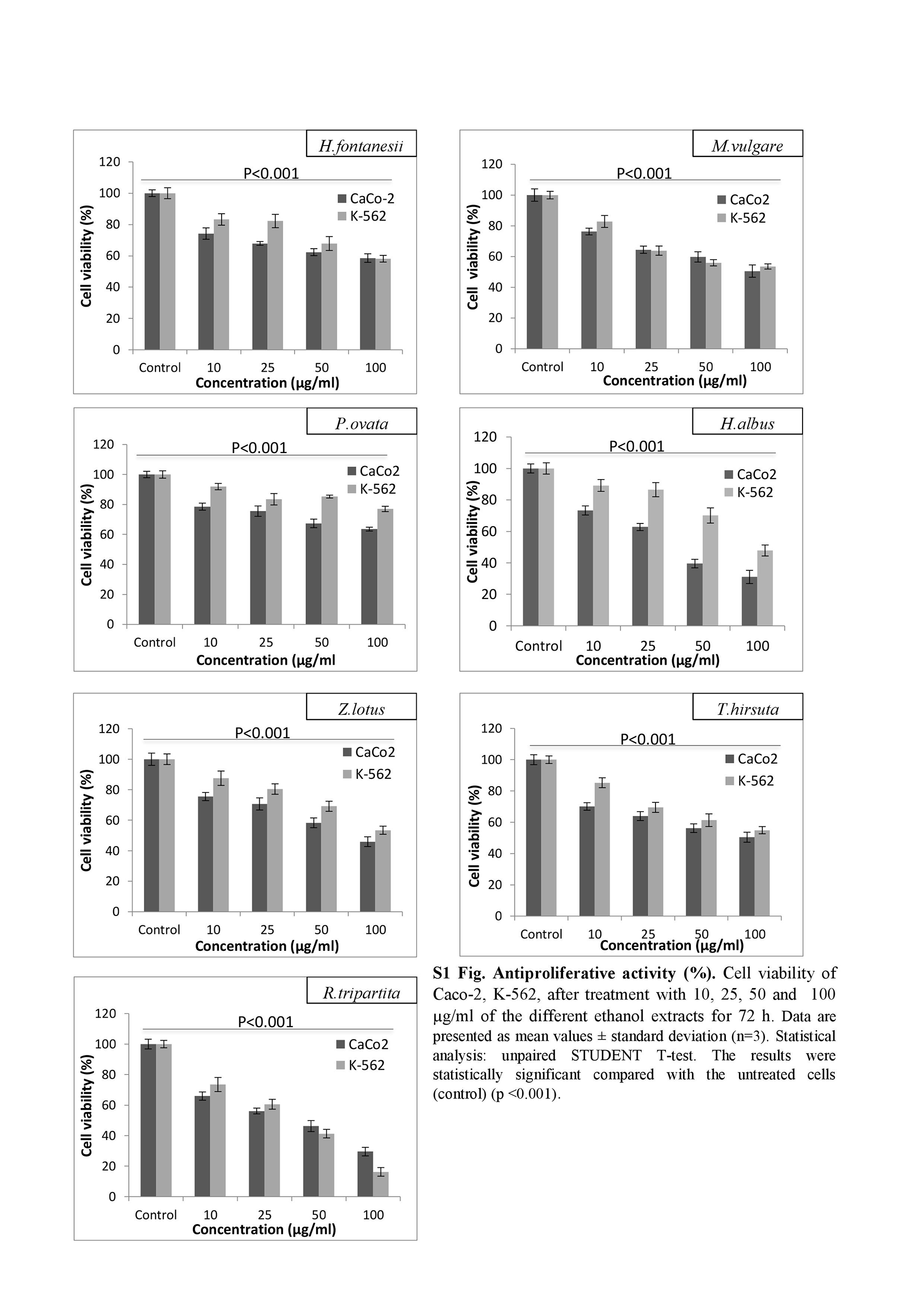

Supplement: S1 Fig — Cell viability of Caco-2, K-562, after treatment with 10, 25, 50 and 100 μg/ml of the different ethanol extracts for 72 h. Data are presented as mean values ± standard deviation (n = 3). Statistical analysis: unpaired STUDENT T-test. The results were statistically significant compared with the untreated cells (control) (p <0.001). (TIF) [file pone.0213049.s001.tif]

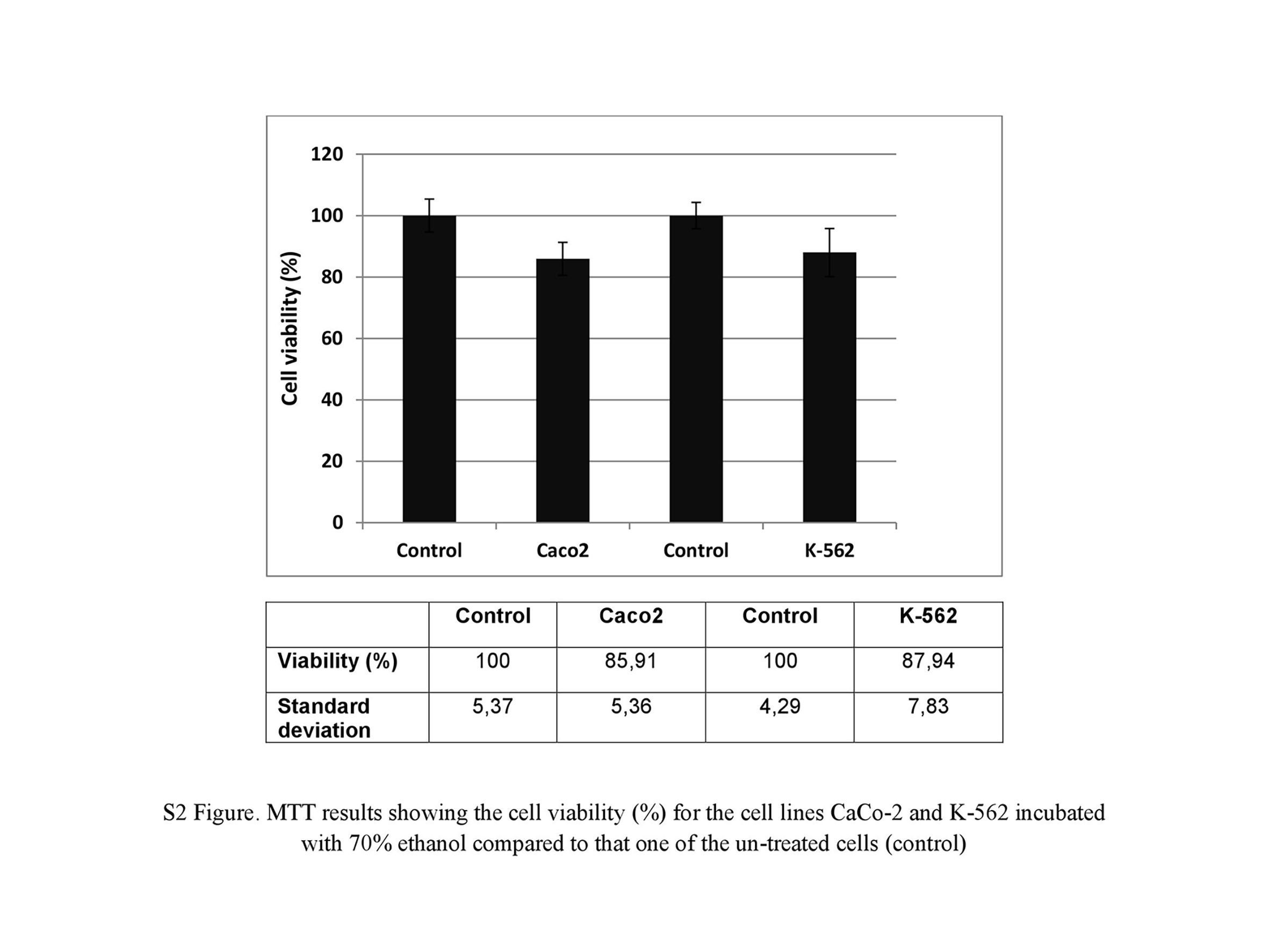

Supplement: S2 Fig — MTT results showing the cell viability (%) for the cell lines CaCo-2 and K-562 incubated with 70% ethanol compared to that one of the un-treated cells (control) (TIF) [file pone.0213049.s002.tif]
